# Three-Dimensional Linear Restoration of a Tunnel Based on Measured Track and Uncontrolled Mobile Laser Scanning

**DOI:** 10.3390/s21113815

**Published:** 2021-05-31

**Authors:** Yulong Han, Haili Sun, Ruofei Zhong

**Affiliations:** Beijing Advanced Innovation Center for Imaging Theory and Technology, Key Laboratory of 3D Information Acquisition and Application, MOE, College of Resource Environment and Tourism, Academy for Multidisciplinary Studies, Capital Normal University, Beijing 100048, China; 2190902205@cnu.edu.cn (Y.H.); zrf@cnu.edu.cn (R.Z.)

**Keywords:** laser scanning, underground space, tunnel linear, 3D point cloud, mobile measurement, track center line

## Abstract

Traditional precision measurement adopts discrete artificial static observation, which cannot meet the demands of the dynamic, continuous, fine and high-precision holographic measurement of large-scale infrastructure construction and complex operation and maintenance management. Due to its advantages of fast, accurate and convenient measurement, mobile laser scanning technology is becoming a popular technology in the maintenance and measurement of infrastructure construction such as tunnels. However, in some environments without satellite signals, such as indoor areas and underground spaces, it is difficult to obtain 3D data by means of mobile measurement technology. This paper proposes a method to restore the linear of the point cloud obtained by mobile laser scanning based on the measured track center line. In this paper, the measured track position is interpolated with a cubic spline to calculate the translations, and the rotation parameters are calculated by combining the simulation design data. The point cloud of the cross-section of the tunnel under the local coordinate system is converted to the absolute coordinate system to calculate the tunnel line. In addition, the method is verified by experiments combined with the subway tunnel data, and the overall point error can be controlled to within 0.1 m. The average deviation in the horizontal direction is 0.0551 m, and that in the vertical direction is 0.0274 m. Compared with the previous methods, this method can effectively avoid the obvious deformation of the tunnel and the sharp increase in the error, and can process the tunnel point cloud data more accurately and quickly. It also provides better data support for subsequent tunnel analysis such as 3D display, completion survey, systematic hazard management and so on.

## 1. Introduction

China has become the world leader in the scale and speed of tunnel and underground engineering construction. The workload of structural deformation and hazard detection of operating tunnels is enormous. For many years, China has faced three recognized problems: “difficulty in measuring comprehensively, difficulty in measuring accurately and difficulty in measuring quickly” [1]. The traditional precise measurement adopts the total-station, artificial static and discrete observation, which cannot meet the demand for large-scale, continuous, dynamic and high-precision measurement in tunnel operation and maintenance management [2].

With the spread of China’s urbanization and the rapid increase in the urban population, the demand for transportation is increasing, and there are more and more subway construction projects in major cities. Subway tunnels have obvious advantages and are widely used in urban construction [3]. A subway is generally narrow in space and complex in environment. A tunnel often deforms under the influence of ground construction facilities and underground construction environment. Subway accidents can cause significant safety hazards and economic losses [4]. Tunnel deformation monitoring needs high precision, high frequency and high timeliness, but at the same time, the environment is complex, the daylight time is short, and the traditional manual operation mode is inadequate for meeting the requirements of subway monitoring [5].

Dynamic detection and monitoring based on laser scanner integration technology is an important developmental direction of tunnel precision measurement. Lidar technology is a new and sophisticated technology gradually developed from the mid-to-late 20th century. It is an active remote sensing technology used to measure the distance between the sensor and the target using the laser emitted by the sensor [6]. Lidar scanning systems can efficiently and quickly obtain the external information of the target and have been widely used in the surveying and mapping industry [7]. According to different scanning methods, it can be divided into station scanning and mobile scanning. In mobile scanning, vehicle-borne mobile measurement technology has become an important technical means to obtain high-resolution spatial information because of its advantages of high precision, high resolution, convenient operation, real-time, nighttime measurement, high efficiency, short mapping cycle, and the continuous and dynamic measurement. Its emergence and development provide a new technical means for the acquisition of three-dimensional spatial information [8].

The 3D scanner is based on the principle of laser ranging. By recording the distance and the horizontal and vertical angles and reflectance, and, based on these elements, by calculating the 3D coordinates of a large number of dense points on the surface of the measured object, it can quickly reconstruct the 3D model of the measured object and various map data such as lines, surface and volume [9,10,11,12]. Mobile laser scanning technology, because of its fast, accurate and convenient measurement advantages, is becoming a popular technology in today’s construction, maintenance, and measurement activities.

Mobile laser scanning usually obtains the point cloud data in a relative coordinate system first, then processes the point cloud data, maintains the linear restoration, and generates the point cloud data in an absolute coordinate system. In some environments with weak or non-existent GPS signals, such as mountain roads, subway tunnels and indoor spaces, it is impossible to obtain 3D information without the help of a high-precision POS system. For this reason, in tunnel engineering, we often abandon the real three-dimensional information and choose the relative measurement method to evaluate the deformation of tunnel structure. As a result, the real three-dimensional space consistency with a horizontal curve and vertical curve is linear, which is not convenient for the integration and sharing of the real space display of results with other structure monitoring or management systems. Therefore, it is necessary to achieve absolute control of relative data.

Data-driven and model-driven methods can be used to locate point cloud data. The traditional precision measurement adopts an artificial static discrete observation and geometric model calculation method, which cannot meet the requirements of large-scale, high-precision, continuous and dynamic measurement in complex conditions such as tracks. In recent years, dynamic observation and dynamic precision measurement of multi-source spatiotemporal data based on multi-sensor integration technology have made great progress in the field of track operation detection and measurement. At present, mobile measurement is generally composed of GNSS and IMU. Its long-term absolute accuracy mainly depends on GNSS, and the accuracy of GNSS is mainly affected by the measurement environment [13,14,15]. In indoor areas and underground spaces, if only relying on INS, the positioning and attitude errors will accumulate rapidly with time, and the error will increase to the upper limit of measurement tolerance in a short time. At this time, how to limit the INS error divergence to maintain a higher absolute accuracy is the main problem of mobile measurement without GNSS.

In recent years, in order to make efficient use of land, a large number of tunnels have been built in Chinese cities, especially as subway projects. In order to carry out inspection, maintenance and construction authorization, it is necessary to carry out accurate measurement of the tunnel [16]. Because GNSS cannot be used in subway tunnel positioning, it is necessary to measure and obtain 3D point cloud data based on control points in the tunnel or design data by integrating a variety of sensors, such as a 3D laser scanner, odometer and gauge-measuring instrument [17,18,19]. Because only the cross-section data under the relative coordinate system are needed for the tunnel clearance measurement, convergence diameter, dislocation deformation detection, etc. [20,21], the Mobile Tunnel Measurement System (MTMS) usually conducts relative measurements on the tunnel scanning data—that is, only the forward direction is expanded according to the mileage, and the actual geometric shape is not restored when the actual linear and geometric position of the tunnel are needed. In general, the point cloud data in a relative coordinate system will be linearly restored.

In the past, the linear restoration of tunnel point cloud data usually directly used the design data method, but there may have been design data inconsistent with the actual construction, or it was difficult to obtain design data due to the age and other reasons, and the data could only be used for display. There are also 3D conformal transformation methods that compare the relative target position obtained by the scanner with the total-station measurement coordinates. In the process of moving laser scanning to obtain the point cloud, the reflector target is pasted at a certain interval on the tunnel wall. At the same time, the total station is used to measure the position of the target center, and the seven-parameter conversion is carried out to obtain the tunnel point cloud data in the absolute coordinate system. This kind of method is not only difficult to implement, but also difficult to manage. Additionally, because of the characteristics of the algorithm, when the measurement distance is long, the tunnel point cloud will produce obvious deformation, and the measurement error will increase sharply. The result is obviously inconsistent with the actual state of the tunnel. We have tried to use the SLAM algorithm for linear restoration; the SLAM algorithm can better adapt to interiors and various multi-featured environments. However, the effect of the application in the repetitive structure environment of a tunnel is not acceptable.

The method we now put forward mainly seeks to process the point cloud data obtained from the measurement in the relative coordinate system, get the point cloud data results in the absolute coordinate system, and carry out three-dimensional display, modeling and digital management. This “3D linear restoration of a tunnel method” is a new name proposed by us, which refers to the restoration of the point cloud in the relative coordinate system to the absolute coordinate system. Similar commercial systems, such as the Leica Sitrack one mobile track scanning system, use the Leica P40 scanner and integrate IMU, GNSS and other positioning systems. Unlike GPS-IMU integrated navigation and other methods, our method is mainly based on the linear restoration of point cloud data in the relative coordinate system from the points on the measured track center line of the tunnel. Integrated navigation is more complex and expensive, but we try to obtain a linear restoration method without additional calculation and cost. In the monitoring of tunnel diameter convergence and staggered platform calculation, the point cloud data results are mainly used and are expanded according to the scanning ring and accurately located according to the mileage. Linear restoration of point cloud data in a relative coordinate system is mainly used for 3D display, modeling and digital management. In the intelligent railway project of a heavy haul railway in China, we use this method for measurement and modeling, which is used for subsequent digital management. At the same time, we compare the point cloud data collected by integrated navigation in this project, and also compare it with the point cloud data collected by airborne radar. This all meets the requirements.

Therefore, this study proposes a method to restore the linear of the point cloud data in a relative coordinate system based on the measured track center line of the tunnel, which combines the method of generating interpolation calculation translations from the measured position with the method of calculating rotation parameters from the simulated design data, and converts the section’s point cloud in the local coordinate system to the absolute coordinate system, so as to calculate the tunnel line. In this paper, based on the MTMS, the point cloud data in the relative coordinate system are restored linearly. According to the measured track center line, the translation parameters are calculated by cubic spline interpolation. The curvature of the track center line is calculated, and the rotation parameters are calculated by dividing different linear and pile positions. The point cloud data in the absolute coordinate system are obtained by linear restoration of the point cloud data in the relative coordinate system through the calculated translation parameters and rotation parameters. This can ensure that the geometry of the tunnel is in line with the actual situation and obtain the actual point cloud coordinate position with high accuracy. It thereby also ensures the accuracy and effectiveness of the tunnel’s section data. Compared with the previous methods, it can effectively avoid the obvious deformation of the tunnel and the sharp increase in the error.

## 2. Linear Restoration Theory

**Figure 1:**
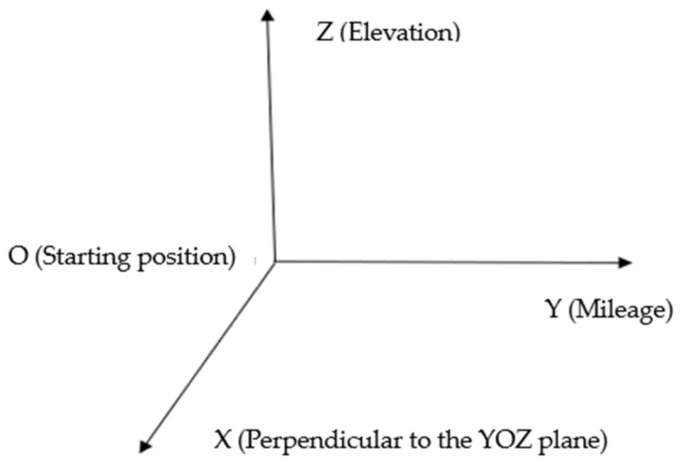
The relative coordinate system of point cloud data.

The point cloud data in the relative coordinate system are usually only expanded according to the mileage in the forward direction, without linear restoration of the actual geometry. As shown in Figure 1, in the relative coordinate system of the point cloud data, the y-axis is the forward direction, that is, the expanded mileage; the z-axis is the height, the x-axis is perpendicular to the YOZ plane, and the x-axis and z-axis take the scanner position as the 0 point.

Linear restoration mainly calculates the track center line through the design data, namely the horizontal curve, superelevation and vertical curve, and then calculates the translation parameters and rotation parameters by comparing each mileage position with the fitting relative track center line, so as to restore the point cloud in the relative coordinate system to the correct position [22].

### 2.1. Design Data Format

#### 2.1.1. Horizontal Curve

A horizontal track curve generally includes a straight line, easement curve and circular curve, in which the straight line and easement curve are connected by a ZH point (point of tangent to spiral) or HZ point (point of spiral to tangent). The easement curve and circular curve are connected by an HY point (point of spiral to curve) or YH point (point of curve to spiral). In this paper, the design data of the horizontal curve are designed according to the format of mileage, X coordinate, Y coordinate, line type, azimuth and radius. In the linear model, 0 represents a straight line, 1 represents a circular curve, and 2 represents an easement curve. Table 1 is an example of horizontal curve.

#### 2.1.2. Vertical Curve

The vertical curve is a section of a curve connecting the turning point of an intersection of two adjacent grade lines on the profile. Unlike the horizontal curve, there are only straight-line and circular-curve segments in the vertical curve, but no easement curve. The vertical curve is generally divided into concave and convex curves. In the convex curve, the overall curve is below the grade-change point, and the radius of the vertical curve is set to a negative value, while in the concave curve, the overall curve is above the grade-change point, and the radius of the vertical curve is set to a positive value. Table 2 is an example of vertical curve.

#### 2.1.3. Superelevation

Superelevation refers to the height difference between two tracks on the vertical plane at the same cross section. In the general curve section, the superelevation is based on the inner track surface, and the outer track is raised to ensure the smoothness of the line. The superelevation of a circular curve segment is constant and is the same as that of the starting point of the curve segment. The superelevation of the straight-line segment is set to 0, so the superelevation of the straight-line segment is also called horizontal. The superelevation of the easement curve is transitional and changes evenly from the beginning to the end. In this paper, the superelevation of the higher side of the track is set as a positive value, and the superelevation of the other side is set as a negative value. The line type of superelevation design refers to the horizontal curve type. Table 3 is an example of superelevation.

### 2.2. Horizontal Linear Calculation

Because the track center points are on the track center line, the line segment between any two main points of the track center line can be regarded as a curve element [23]. When the horizontal design curve data of the track are obtained, the plane coordinates (*X_P_*, *Y_P_*) of any point P on the curve element and the corresponding tangent azimuth *α_P_* can be obtained from the Gauss Legendre formula in the numerical integration.

(1) $$X_{P}=X_{A}+l\overset{4}{\underset{i=1}{\sum }}R_{i}\;\cos[\alpha_{A}+c(K_{A}V_{i}l+\frac{\left(K_{B}-K_{A}\right)V_{i}^{2}}{2L_{S}}l^{2})]$$

(2) $$Y_{P}=Y_{A}+l\overset{4}{\underset{i=1}{\sum }}R_{i}\;\sin[\alpha_{A}+c(K_{A}V_{i}l+\frac{\left(K_{B}-K_{A}\right)V_{i}^{2}}{2L_{S}}l^{2})]$$

(3) $$\alpha_{P}=\alpha_{A}+c(K_{A}V_{i}l+\frac{\left(K_{B}-K_{A}\right)V_{i}^{2}}{2L_{S}}l^{2})$$

In Equations (1)–(3), (*X_P_*, *Y_P_*) are the plane coordinates of the starting point A on the curve element AB, and B is the end point; *K_A_* and *K_B_* are the curvature of point A and point B, respectively; *K_AB_* = *K_A_* − *K_B_*; *L_P_* is the mileage of point P; *α_A_* is the azimuth of point A; *L_S_* is the arc length of the bending element; *α_P_* is the azimuth of any point P on the curve element ab; *c* is the bending direction of the curve (when the curve bends to the right, *c* = +1; when the curve bends to the left, *c* = −1); and *R_i_* and *V_i_* are constants, which are integral coefficients and integral nodes, respectively.

(4) $$\begin{array}{c} R_{1}=R_{2}=0.1739274226;\\ R_{3}=R_{4}=0.3260725774;\\ V_{1}=0.0694318442;\;V_{2}=0.330009478;\\ V_{3}=0.6699905218;\;V_{4}=0.9305681558; \end{array}$$

### 2.3. Vertical Linear Calculation

The vertically designed curve can be used to calculate the elevation of the center point in the coordinate system of the design route. When the measured section is located on the straight section of the vertical curve, the elevation of the track center point can be directly interpolated according to the length of the curve, the elevation of the starting and ending points, and the measured section mileage according to the mileage ratio. If the measured section is located in the circular curve segment of the vertical curve, the center coordinates (*Mo*, *Ho*) of the circular curve are obtained by coordinate transformation, and then the elevation *H_P_* of the track center point P is calculated by taking the distance between the center of the circular curve and any point P on the curve as the radius R.

The vertically designed curve can be used to calculate the vertical deflection angle of the tunnel section. If the section is in the straight section of the vertical curve, the vertical deflection angle is 0°. If the section is in the circular curve section of the vertical curve, its vertical deflection angle is the slope value in the line coordinate system. In the mileage elevation coordinate system, the curve element solution model is used to calculate *α_M_*.

(5) $$\left(\begin{array}{c} M_{o}\\ H_{o} \end{array}\right)=\left(\begin{array}{c} Mile\\ H \end{array}\right)+\left(\begin{array}{c} \cos\left(\pi +i\right)-\sin\left(\pi +i\right)\\ \sin\left(\pi +i\right)-\cos\left(\pi +i\right) \end{array}\right)\left(\begin{array}{c} T\\ R \end{array}\right)\;$$

(6) $$H_{P}=-k*\sqrt{R^{2}-{\left(M_{p}-M_{o}\right)}^{2}}+H_{o}$$

(7) $$\alpha_{M}=\alpha_{P}+c\left(K_{A}l+\frac{\left(K_{B}-K_{A}\right)}{2L_{S}}l^{2}\right)\;$$

## 3. Linear Restoration Method

In this paper, the method of linear restoration of the tunnel point cloud is mainly based on the point cloud data in the relative coordinate system generated by the MTMS. In the past, the linear restoration of tunnel point cloud data was usually directly based on the design data method. However, due to the long history, tunnel deformation and other problems, it may be difficult to obtain the design data from the actual construction. The results show that the restored tunnel linear is obviously inconsistent with the actual state. Therefore, this paper proposes a method to automatically calculate the position and angle of the tunnel section according to the measured track centerline interpolation and curvature calculation, and then restore the tunnel point cloud data in an absolute coordinate system.

In the experiment, the total station is used to measure the points on the track’s center line. In the survey, after the total station is set up, the point is led from the pile control network (CPIII) for surveying. The pile control network (CPIII) is the most basic control network in the railway survey [24,25]. For the three-dimensional control network arranged along the line, the plane control starts and closes at the basic plane control network (CPI) or the line control network (CPII); the elevation control starts and stops at the second-class leveling network arranged along the line. Generally, the survey is carried out after the completion of the offline engineering construction, which is the benchmark for track laying and operation and maintenance proposed by the Ministry of Railways of the People’s Republic of China. CPIII is the track control network, which mainly provides the control benchmark for track laying and operation and maintenance. The subgrade is also set with a point every 100 m or so on the electrified pole base on both sides of the lower and upper sides. The CPIII plane control survey adopts free station side angle intersection method, and elevation control network observation adopts the one-way precise leveling method.

Thus, in the point cloud in the relative coordinate system, the relationship between the track center and the relative position of the scanner can be regarded as a constant; that is, the relative track center of each mileage section can be obtained by calibrating the X and Z coordinates. According to the design data, we calculate the design of the track center line or directly use the measured track center line data to obtain the three-dimensional coordinates of each mileage section track-center point, so as to calculate the translation parameters [26]. In the point cloud in the relative coordinate system, the values for three directions of each section are zero. Therefore, as long as the three attitude angles corresponding to each section in the design line are calculated, namely the plane azimuth angle, vertical deflection angle and lateral inclination angle, the rotation parameters can be calculated.

**Figure 2:**
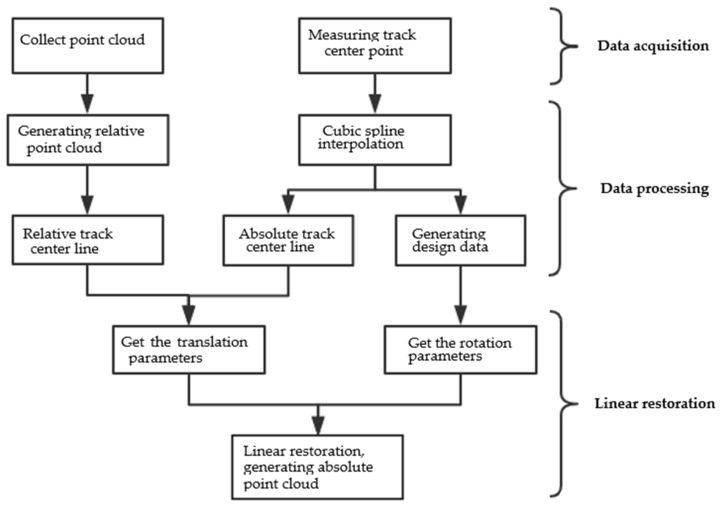
Flow chart of restoration of the measured centerline linear.

Because the complexity of the horizontal curve linear is higher than that of the vertical curve linear, there is an easement curve between the straight line and circular curve [27]. In this paper, the linear fitting with the straight-line segment, easement-curve segment and circular-curve segment is mainly used for calculation and verification. Specifically, in this paper, the measured track center point of the spline curve is used for cubic spline interpolation to obtain the track center line, which is connected with the track center line in the relative coordinate system to calculate the translation parameters; the curvature of the track center line is calculated to determine each linear section and pile point; according to the linear and pile position, the horizontal deflection angle, lateral inclination angle and vertical deflection angle can be calculated. These angles constitute rotation parameters. According to the translation parameters and rotation parameters, the actual point cloud position can be restored. The data processing flow of the method of line restoration is shown in Figure 2.

### 3.1. Translation Parameters

The coordinate calculation of the track center in the car body coordinate system can obtain the coordinate of the section center from the fitting calculation of the point cloud section, and then get the translation parameters from the scanner center to the track center by checking the car, and get the offset of the track center relative to the section center from the first two steps, so as to calculate the coordinate of the track center in the car body coordinate system. The relative track center line is determined according to mileage.

**Figure 3:**
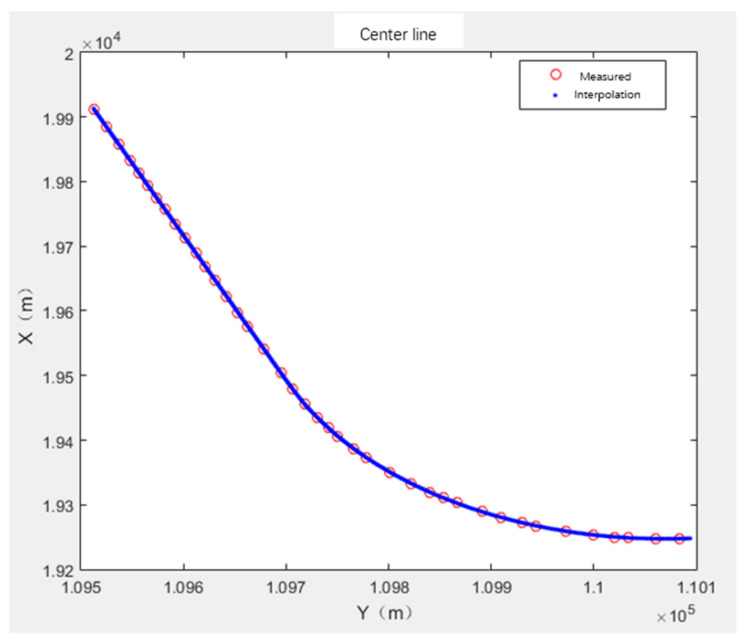
Interpolation center line of measured data.

The measured track center line uses the total station to measure the track center point coordinates and corresponding mileage. The plane coordinates of the track center point at each mileage are obtained by spline interpolation of the measured track center X and Y coordinates, as shown in Figure 3. The track center point elevation at each mileage is obtained by spline interpolation of the mileage M and elevation H coordinates, and the translation parameters can be obtained by comparing this with the relative center line generated by fitting.

### 3.2. Curvature Parameters

**Figure 4:**
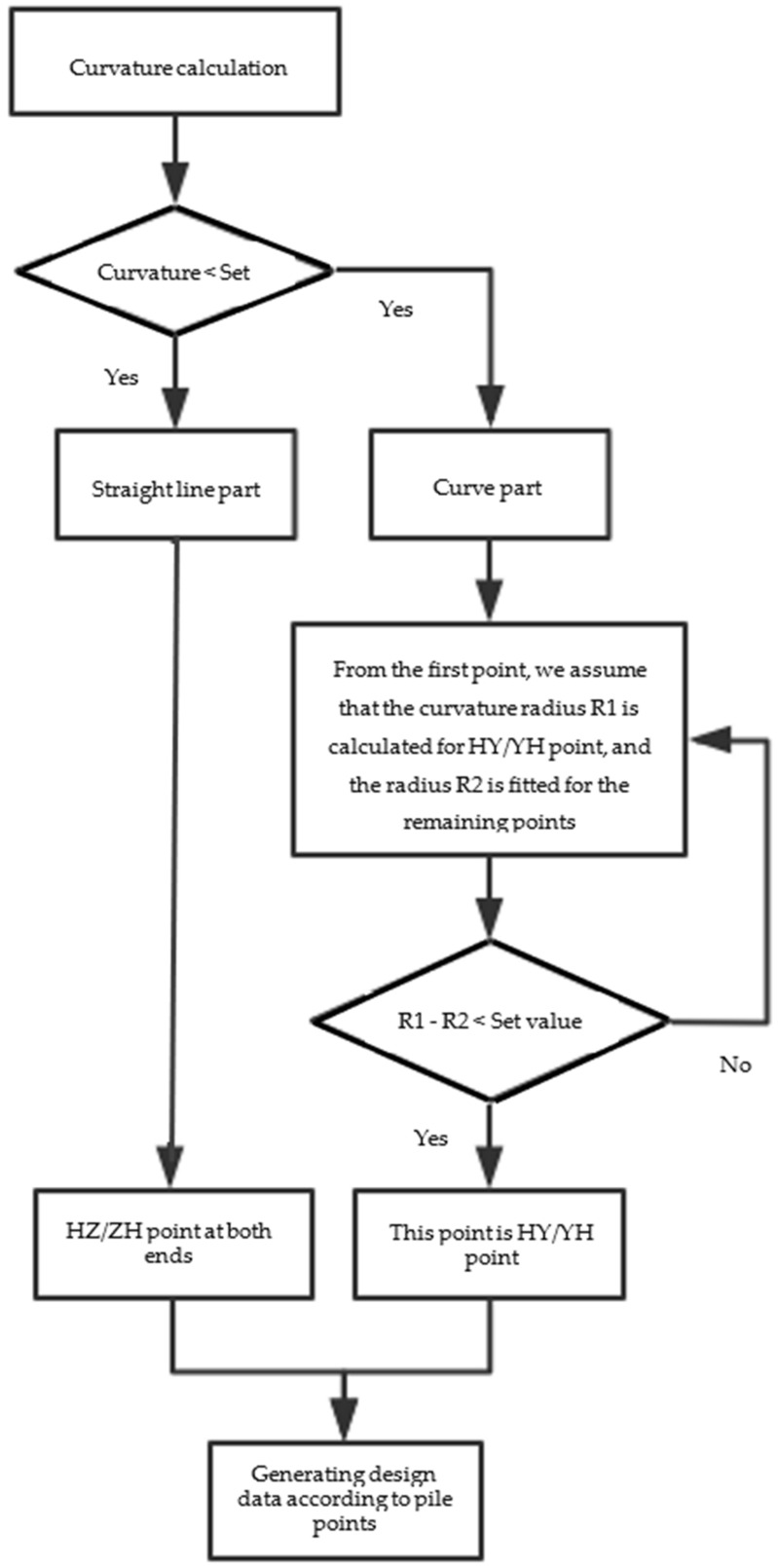
Flow chart of design data generated by curvature calculation.

The curvatures of each track center line generated by interpolation are calculated to judge each linear section. Generally, the radius of the urban transit track curve will not exceed 5000 km—that is, the curvature is greater than 0.0002. According to the measured data, the part with curvature less than 0.0001 is regarded as the straight part, and the part with curvature more than 0.0001 is regarded as the curved part; for the curved part, according to the curvature and mileage, the fitting radius of the circular curve and the curvature radius of the HY point are calculated point by point. When the two are equal, they are the HY points. The processing flow is shown in Figure 4.

For the center line of the track generated by interpolation, the curvature is calculated as follows:

(8) $$K=\frac{\left|y^{\prime \prime }\right|}{{\left(1+y^{\prime }{}^{2}\right)}^{\frac{3}{2}}}$$

**Figure 5:**
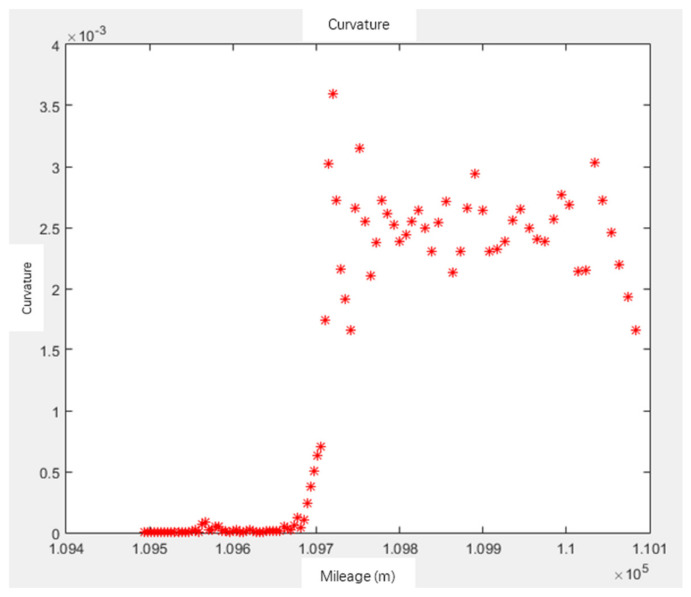
Curvature calculation.

**Figure 6:**
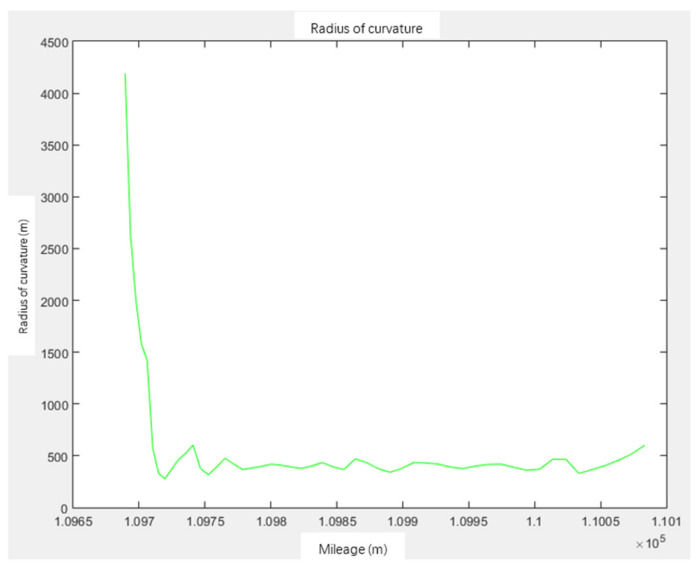
Radius of curvature.

The curvature graph and curvature radius are obtained as shown in Figure 5 and Figure 6. The curvature of the line K=0; the curvature of the circular curve is the reciprocal of the design radius R of the circle, K=1/R; the easement curve is maintained at $RL=A^{2}$ ($A^{2}$ is a constant), where L is the mileage from the starting point of the easement curve; that is, $K=1/R=L/A^{2}$. So, curvature K is a function of mileage L, that is, $K=f\left(L\right)$ [28].

**Figure 7:**
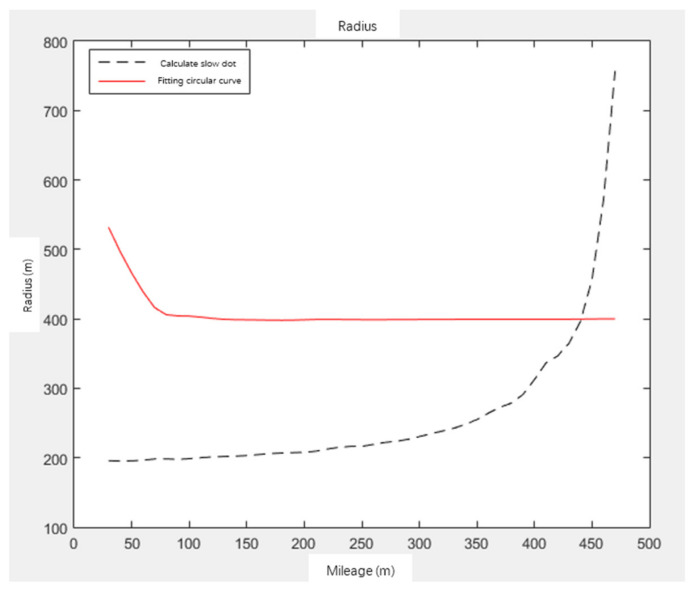
Fitting radius of circular curve R_1_ and radius of HY point R_2_.

**Figure 8:**
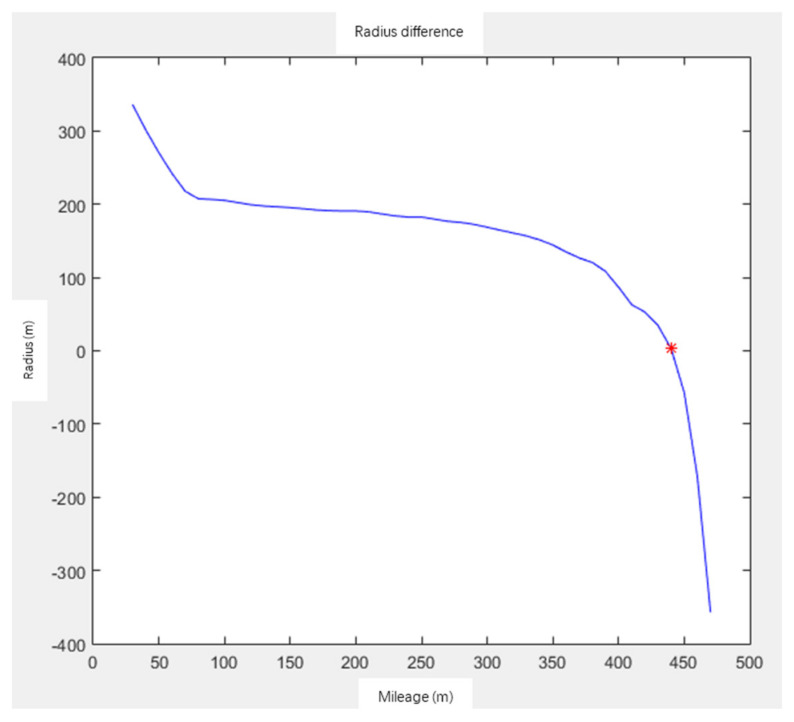
Radius difference of each point.

For the calculation of the coordinates of HY points on the curve, according to the curvature K and mileage L, assuming that each mileage is taken as the HY points, the fitting radius $R_{1}$ of the circular curve is calculated, and the parameter $A=\sqrt{L/K}$ of the gentle curve is calculated, as well as the curvature radius $R_{2}$ of the HY points under this parameter. When $d_{R}=R_{1}-R_{2}$ is equal to 0, it is an HY point. The calculation of HY points is shown in Figure 7 and Figure 8. When the fitting radius of the circular curve is equal to the curvature radius of HY points, it is the HY point.

### 3.3. Rotation Parameters

According to the location of the linear section, pile point and radius of the circular curve, the simulation design data can be generated, and the rotation parameters of each mileage section can be calculated.

#### 3.3.1. Horizontal Deflection Angle

Because the track center points are on the track center line, the line segment between any two main points of the track center line can be regarded as a curve element. After obtaining the data of the simulated design of the horizontal curve, the azimuth *α_P_* of each track center point P on the curve element can be calculated by the Gauss Legendre formula.

(9) $$\alpha_{P}=\alpha_{A}+c(K_{A}V_{i}l+\frac{\left(K_{B}-K_{A}\right)V_{i}^{2}}{2L_{S}}l^{2})$$

In Equation (9), *K_A_* and *K_B_* are the curvature of point A and point B, respectively; *l* is the mileage of point P; *α_A_* is the azimuth of point A; *L_S_* is the arc length of the element; *α_P_* is the azimuth of any point P on the curve element ab; *c* is the bending direction of the curve (when the curve bends to the right, *c* = +1; when the curve bends to the left, *c* = −1); and *R_i_* and *V_i_* are constants and are integral coefficients and integral nodes, respectively.

#### 3.3.2. Lateral Inclination

**Figure 9:**
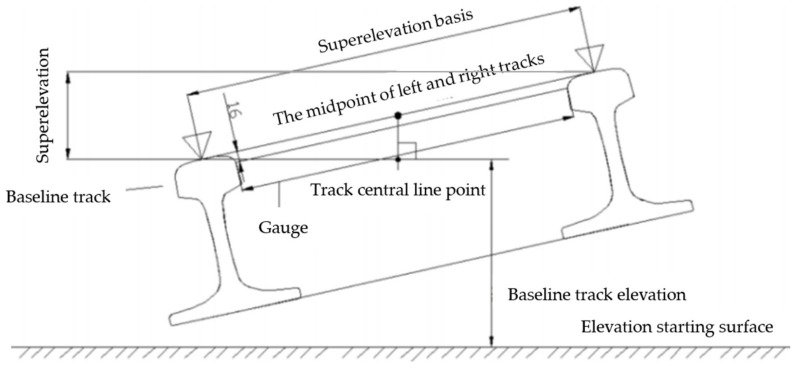
Schematic diagram of the track geometric parameters.

Superelevation and lateral inclination refer to horizontal curve design data, which can also be divided into a straight line, easement curve and circular curve. The superelevation of the straight-line segment is 0, the superelevation of the circular curve segment is constant and the superelevation value is the same as that of the starting point of the curve segment, while the superelevation of the eased curve segment changes uniformly from the starting point to the end point. The lateral inclination is the ratio of superelevation-to-superelevation datum, as shown in Figure 9.

The calculation Formula (10) of the superelevation is as follows:

(10) $$c_{h}=11.8\frac{V^{2}}{R}$$

where *V* is the design speed; *R* is the curvature radius of the horizontal curve.

In the actual calculation, according to the mileage data of the measured section, the section of the superelevation line corresponding to the track center of the measured section can be determined. The superelevation angle of each point on the curve can be calculated through the superelevation of the starting point and the ending point of each curve section, and the calculation method is different according to the curve type [29]. For the straight section, the superelevation is always 0; the superelevation of the circular curve section is constant and is the same as the superelevation of the starting point of the section. If a section is located in a circular curve segment, its track inclination angle *α* can be calculated according to the superelevation datum and the superelevation constant of the section

(11) $$\alpha_{\gamma }=arc\sin(\frac{c_{h}}{c_{d}})$$

According to Formula (12), the track lateral inclination of a point on the easement curve section can be calculated:

(12) $$\alpha_{H}=arc\tan(\frac{\tan\;\alpha_{0}}{L_{S}}L_{1})$$

In Formula (12), *c_d_* is the superelevation datum, generally 1500 mm, *L_S_* is the total length of the easement curve, and *L*_1_ is the mileage difference between the mileage of the track center point to be calculated and the starting point of the easement curve section, where $\frac{\tan\;\alpha_{0}}{L_{S}}$ is the tangent proportion constant of the track’s lateral inclination in the easement curve section.

#### 3.3.3. Vertical Deflection Angle

The vertical deflection angle of the tunnel section can be calculated from the simulated vertically designed curve. If the section is in the straight section of the vertical curve, the vertical deflection angle is 0°. If the section is in the circular curve section of the vertical curve, its vertical deflection angle is the slope value in the line coordinate system. In the mileage elevation coordinate system, the curve element solution model is used to calculate *α_M_*:

(13) $$\alpha_{M}=\alpha_{P}+c(K_{A}l+\frac{\left(K_{B}-K_{A}\right)}{2L_{S}}l^{2})$$

## 4. Case Study

### 4.1. Experimental Equipment

**Figure 10:**
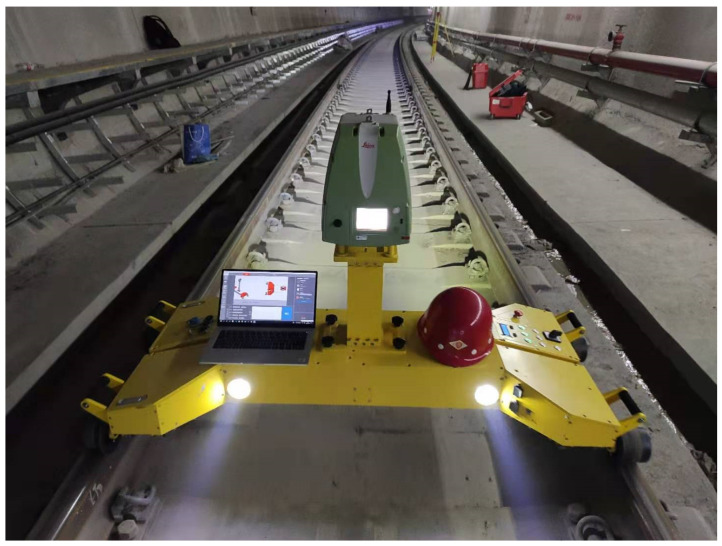
Mobile Tunnel Measurement System (MTMS).

The MTMS used in this test mainly integrates a variety of sensors, such as the three-dimensional laser scanner, non-contact odometer and gauge measuring instrument, as shown in Figure 10. The MTMS can obtain high-quality three-dimensional point cloud data and accurate mileage without a GNSS signal. The detection trolley moves forward at a constant speed.

The Leica ScanStation P16 professional engineering scanner is selected for data acquisition in this test, and its model and main technical parameters are as follows. In this scanning experiment, the speed of the detection car is about 1.5 m/s, and the scanner speed is 100 Hz. The radial density of the point cloud is about 15 mm, and the circumferential density is about 1 mm.

The Leica 3D laser scanner used in the project is the Leica ScanSation P16 professional engineering scanner, and its main technical parameters are shown in Table 4:

### 4.2. Experimental Overview

The experimental data of this paper were used in the Shenzhen Metro, and the Leica ScanSation P16 scanner was used to scan the shield tunnel. In addition, the MTMS was used to verify the research method proposed in this paper.

**Figure 11:**
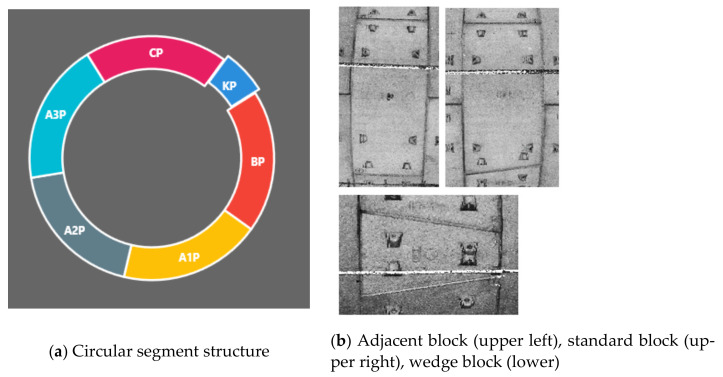
Structural drawing of shield tunnel.

The shield tunnel in this section was assembled in staggered joints; Figure 11 shows the structure of the tunnel segment. The ring width of the lining was 1.5 m and the inner diameter of the segment was 5.4 m. There are three types of curves: circular curve, easement curve and straight section. In order to test the accuracy, a number of targets were pasted in the measurement section of the tunnel detection car, and the total station was used to measure the target coordinates. Finally, the target coordinates in the point cloud data in the absolute coordinate system generated by linear restoration were compared with the total station coordinates.

**Figure 12:**
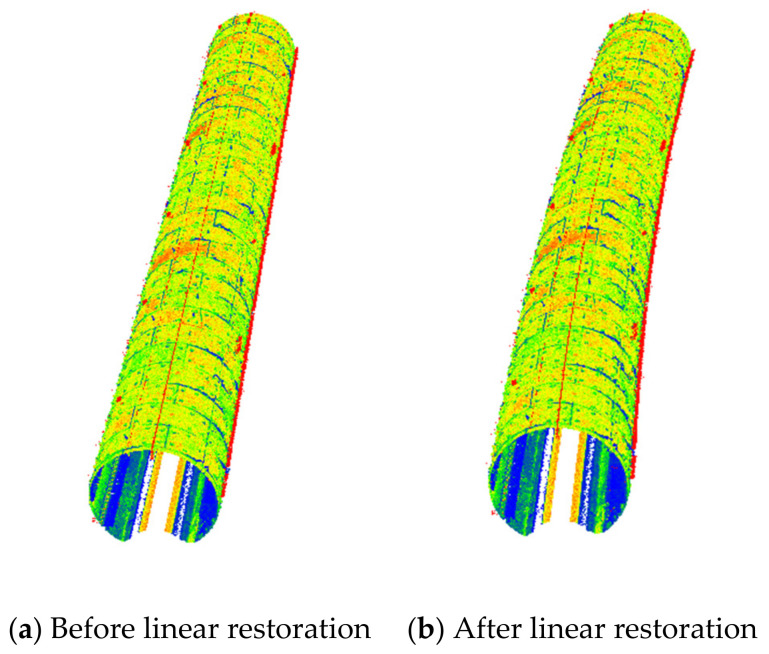
Comparison results of tunnel linear before and after reconstruction.

**Figure 13:**
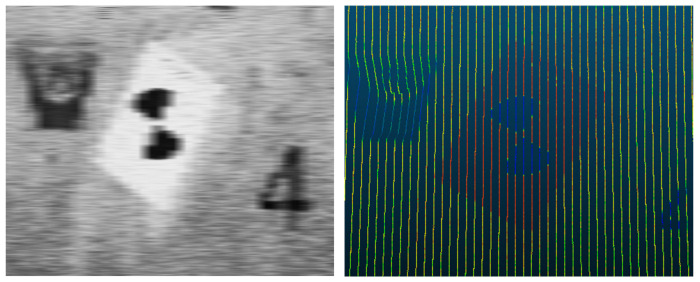
Point cloud extraction target.

The comparison diagram before and after linear restoration is shown in Figure 12, and Figure 13 shows the target in the point cloud. Figure 12 shows the comparison of point cloud data before and after linear restoration. The color in the image represents the intensity information of the scanning point cloud. Before the restoration, the point cloud data have a straight barrel shape, only along the mileage, and there is no real geometric change; the restored point cloud data demonstrate the real position in the absolute coordinate system, which shows the real geometry of the tunnel. Figure 13 displays the target image and point cloud to verify the conversion accuracy. The color in the left image is the gray image of the scanning data, and the color in the right image represents the intensity information of the scanning point cloud. The accuracy is verified by comparing the errors between the coordinates measured by the total station and the coordinates of the point cloud after linear restoration. The target is pasted directly on the wall of the tunnel and evenly pasted on the left and right sides of the tunnel according to the mileage.

### 4.3. Point cloud Restoration Accuracy

Generally, tunnel detection requires very strict accuracy. In China, this is done according to the *Specification for construction and acceptance of shield tunnels* (GB 50446-2017). For example, in subway tunnel, the ovality limit of lining ring is ±6‰, the step limit of the inner ring is 10 mm, and the step limit of inter ring is 15 mm. So, the measurement of section convergence, the wrong platform and other point cloud data in the relative coordinate system must reach millimeter-level accuracy [30]. According to the experimental data, we get the results of convergence diameter detection and the calculation results of dislocation. For the point cloud data converted from the relative coordinate system to the absolute coordinate system by linear restoration, it is generally used for 3D modeling, digital management and other purposes, and the current accuracy is usually centimeter-level.

The accuracy of linear restoration is mainly obtained by comparing the coordinates of the target placed in the tunnel after linear restoration with the coordinates measured by the total station. In order to test the linear restoration accuracy, multiple targets are placed in the scanning section, and the target coordinates are measured by the total station. The target coordinates in the point cloud after linear restoration are compared with the coordinates measured by the total station to calculate the error. Table 5 shows the target coordinates measured by the total station. Table 6 shows the errors.

It can be seen from Table 6 that the maximum deviation of the measured centerline is −79.6 mm, with an average deviation of 40.4 mm, the maximum deviation of the Y axis is −85.0 mm, with an average deviation of 26.2 mm, and the maximum deviation of the Z axis is −94.9 mm with an average deviation of 27.4 mm. It can be seen that the error of the measured centerline method is small.

The point position error can be controlled to within 0.1 m with the center line restoration method. After the linear restoration of the measured centerline method, the maximum deviation of the horizontal direction point is 87.5 mm, with an average deviation of 55.1 mm, and the maximum deviation of the vertical direction is −94.9 mm, with an average deviation of 27.4 mm. In this paper, the 3D linear reconstruction of the tunnel is maintained, and the actual high-precision point cloud coordinate data are obtained. It also ensures the accuracy and effectiveness of the tunnel section data, which provides more data support for the future tunnel analysis.

## 5. Discussion

The method we put forward at this time mainly consists of processing the point cloud data obtained from the measurement in the relative coordinate system, in order to obtain the point cloud data results in the absolute coordinate system, and to carry out three-dimensional display, modeling and digital management [31]. At present, the accuracy of this method is at the centimeter level, which needs to be further improved. In a section of the advanced railway project of a heavy-haul railway in China, we use this method to measure and model for the subsequent digital management. We use the airborne scanning data of the same section of the railway tunnel entrance for comparison. This section of railway is a heavy haul railway which has been running for decades, and the tunnel is relatively large. The actual project requires that the accuracy of 3D coordinate plane and elevation of the tunnel is within 20 cm. The method and the measured data verify that it meets the requirements of the project.

In addition, in the process of linear restoration of point cloud in the relative coordinate system, the simulation design data can be obtained based on the measured data, which can be used in the calculation of track regularity. In the case of no design data or inaccurate original design data, the simulation design data can be used to calculate the track regularity. After improving the accuracy, this method may also be used for 3D reconstruction of tunnel scenes [32,33]. At present, the methods of 3D scene reconstruction using the collected 3D point cloud data are mainly based on feature point registration or GNSS/IMU integrated navigation to obtain the attitude and position. However, there is no good method to deal with the point cloud data obtained by tunnel moving scanning without GPS signal. We will strive to improve the accuracy, and try to combine this method with the characteristics of repetitive structures such as tunnel segments and railway sleepers to achieve 3D scene reconstruction in this case.

In the next step, we will explore the installation of the inertial navigation module, supplemented by a small number of control points to correct the inertial navigation data, so as to update and calculate the position and attitude more conveniently and efficiently [34,35]. Additionally, we will explore the application of this method in other scenarios. On the other hand, we will also try to improve the SLAM algorithm in order to improve the application effect of the tunnel in this repetitively structured environment [36,37].

## 6. Conclusions

This method is based on the measured track center line of the tunnel, uses the correlation algorithm, generates interpolation according to the measured position to calculate the translations, and combines with the method of calculating the rotation parameters through the simulation of design data; then, the position and angle of the tunnel section are calculated automatically; finally, the cross-section point cloud in the local coordinate system is converted to the absolute coordinate system, and the true alignment of the tunnel is restored, and the tunnel point cloud data in the absolute coordinate system are obtained.

The point error of the proposed method can be controlled to within 0.1 m; the average deviation of the horizontal point is 55.1 mm, and the average deviation of the elevation direction is 27.4 mm.

In the past, we usually used the design data to restore the point cloud in the relative coordinate system linearly. This time, we propose a new method to restore the point cloud in the relative coordinate system linearly by using the measured track center line, which is different from the method of using the design data directly. We do not need the aid of integrated navigation and other auxiliary systems. We have not found any other similar method. This method can greatly reduce the cost of obtaining a point cloud in absolute coordinate system, and has great significance in 3D modeling, digital management and other applications. It can provide better data support for transit tunnel track 3D displays, 3D reconstruction [38], completion survey, systematic hazard management [39] and other follow-up tunnel analysis and management, and can also provide a good perspective for future research. At the same time, the track measurement data used in this method can be combined with the tunnel maintenance to obtain the measured data of the center line directly from the engineering department, so as to avoid increasing the measurement workload.

## Figures and Tables

**Table 1:** Example of horizontal curve design.

Mileage (m)	X (m)	Y(m)	Line Type	Azimuth (rad)	Radius (m)
430.1700	109,730.532	19,434.9886	1	2.146360499	400
587.4350	109,662.5493	19,576.6594	2	1.990945913	400
786.2070	109,581.5084	19,758.1604	0	1.990945913	0

**Table 2:** Example of vertical curve design.

Mileage (m)	Elevation (m)	Radius (m)
26,640.0000	475.222	−5000
27,140.0000	484.222	−5000
27,400.0000	490.982	3000

**Table 3:** Example of superelevation design.

Mileage (m)	Left Offset (m)	Left Superelevation (m)	Right Offset (m)	Right Superelevation (m)	Line Type
430.1700	−0.75	−0.06	0.75	0.06	1
587.4350	−0.75	−0.06	0.75	0.06	2
786.2070	−0.75	0	0.75	0	0

**Table 4:** Main parameters of the Leica ScanStation P16 scanner.

Item	Parameter
Vertical view	290°
Horizontal view	360°
Maximum rate	1000 thousand/s
Ranging error	1.2 mm + 10 ppm (within range)
Scanning range and reflectivity	80 m; 18% (minimum distance 0.4 m)

**Table 5:** Target coordinates measured by total station.

Name	Relative Mileage Position	Target Coordinates (Control Points)		
		Y(N) (m)	X(E) (m)	Z(H) (m)
D1	86.281	109,567.315	19,783.415	16.152
D2	145.296	109,596.257	19,731.646	16.089
D3	194.581	109,611.476	19,684.407	14.278
D4	245.073	109,636.998	19,640.374	14.509
D5	296.096	109,653.077	19,591.579	12.605
D6	336.396	109,674.376	19,556.818	12.771
D7	495.558	109,742.521	19,412.725	9.140
D8	596.837	109,811.873	19,338.059	7.560
D9	647.219	109,855.657	19,312.479	7.696
D10	698.532	109,898.073	19,283.051	5.854
D11	748.202	109,946.278	19,269.605	5.944
D12	797.422	109,992.631	19,252.118	4.293
D13	849.228	110,044.753	19,250.775	4.182

**Table 6:** Target error of restoration point cloud extraction.

Name	Restoration Deviation Value of Measured Center Line			
	dY (mm)	dX (mm)	Horizontal dl (mm)	Elevation dZ (mm)
D1	−16.3	−21.3	26.8	−12.9
D2	7.1	4.1	8.2	37.1
D3	17.6	12.9	21.8	29.4
D4	−6.3	45.4	45.9	25.7
D5	−23.2	58.1	62.6	−18.2
D6	−8.5	73.2	73.7	5.6
D7	2.2	−24.4	24.5	−94.9
D8	8.4	−79.6	80	−52.1
D9	−73.5	−24.1	77.3	38.6
D10	−7	−78.4	78.7	−10.3
D11	−85	−20.6	87.5	26.5
D12	39	−70.1	80.2	−0.1
D13	47	−13.1	48.8	4.5
Absolute average	26.2	40.4	55.1	27.4

## Data Availability

Not report any data.
